# Associations and dynamics of Vibrionaceae in the environment, from the genus to the population level

**DOI:** 10.3389/fmicb.2014.00038

**Published:** 2014-02-11

**Authors:** Alison F. Takemura, Diana M. Chien, Martin F. Polz

**Affiliations:** Parsons Lab for Environmental Science and Engineering, Department of Civil and Environmental Engineering, Massachusetts Institute of TechnologyCambridge, MA, USA

**Keywords:** *Vibrio*, population, environmental correlation, ecology, niche, attachment, planktonic

## Abstract

The Vibrionaceae, which encompasses several potential pathogens, including *V. cholerae*, the causative agent of cholera, and *V. vulnificus*, the deadliest seafood-borne pathogen, are a well-studied family of marine bacteria that thrive in diverse habitats. To elucidate the environmental conditions under which vibrios proliferate, numerous studies have examined correlations with bulk environmental variables—e.g., temperature, salinity, nitrogen, and phosphate—and association with potential host organisms. However, how meaningful these environmental associations are remains unclear because data are fragmented across studies with variable sampling and analysis methods. Here, we synthesize findings about *Vibrio* correlations and physical associations using a framework of increasingly fine environmental and taxonomic scales, to better understand their dynamics in the wild. We first conduct a meta-analysis to determine trends with respect to bulk water environmental variables, and find that while temperature and salinity are generally strongly predictive correlates, other parameters are inconsistent and overall patterns depend on taxonomic resolution. Based on the hypothesis that dynamics may better correlate with more narrowly defined niches, we review evidence for specific association with plants, algae, zooplankton, and animals. We find that *Vibrio* are attached to many organisms, though evidence for enrichment compared to the water column is often lacking. Additionally, contrary to the notion that they flourish predominantly while attached, *Vibrio* can have, at least temporarily, a free-living lifestyle and even engage in massive blooms. Fine-scale sampling from the water column has enabled identification of such lifestyle preferences for ecologically cohesive populations, and future efforts will benefit from similar analysis at fine genetic and environmental sampling scales to describe the conditions, habitats, and resources shaping *Vibrio* dynamics.

## Introduction

The family *Vibrionaceae* (or vibrios for short) comprises a genetically and metabolically diverse group of heterotrophic bacteria that are routinely found in all ocean environments, ranging from coastal to open and surface to deep water (Thompson et al., 2004; Thompson and Polz, 2006). Moreover, a few *Vibrio* species have extended their range beyond the marine environment, occurring predominantly in brackish and even freshwater environments (Thompson et al., 2004). The study of the environmental distribution and dynamics of vibrios has a long history, largely because many species contain potential human and animal pathogens (Thompson et al., 2004, 2005). Hence there is considerable public health and economic interest in determining factors correlated to increased abundance of vibrios (Stewart et al., 2008). Moreover, vibrios are easily cultured on standard and selective media and thus were highly visible in the pre-molecular era of microbial ecology. In recent years, environmental dynamics have also been studied with culture-independent methods allowing for a more fine-scale assessment of environmental drivers of occurrence, and the vibrios have become a model for bacterial population biology and genomics. In fact, presently, the vibrios represent one of the best-studied models for the ecology and evolution of bacterial populations in the wild.

The early discovery that some fish species harbor high numbers of vibrios (e.g., Liston, 1954, 1957; Aiso et al., 1968; Sera et al., 1972) has led to the widespread notion that these bacteria are only transient members of microbial assemblages of the water column. Instead, vibrios were regarded as specifically associated with animals, and occurrence in water samples was thought to be primarily due to their excretion with fecal matter. This picture was enforced by the discovery that several luminescent *Vibrio* (*Allivibrio*) and related *Photobacterium* species form intimate symbioses with animals (e.g., fish, squid) (Ruby and Nealson, 1976; Stabb, 2006). More recent work has, however, revealed that the notion of vibrios being “enterics of the sea” (Liston, 1954) represents an oversimplification. Many *Vibrio* species grow actively in ocean water either in the free-living phase or associated with various types of organic particles, many of which are of non-animal origin (Lyons et al., 2007; Froelich et al., 2012). Thus although association with animals can be an important part of the life cycle of many *Vibrio* species, there are others that only loosely associate with animals or not at all, an aspect we explore in detail in this review.

Another widely held belief about vibrios is that they play a relatively minor role in chemical transformations in the ocean, despite the wide range of metabolisms [e.g., chitin degradation (Hunt et al., 2008a; Grimes et al., 2009)] of which they are capable. This belief is largely based on low to medium average relative abundance of *Vibrionaceae* in ocean water. Yet three considerations suggest that the role of vibrios has been underestimated. First, it has been pointed out that although vibrios' abundances are generally only around 10^3^ to 10^4^ cells per ml seawater (i.e., on the order of few percent of total bacteria), they have very high biomass (Yooseph et al., 2010). For example, an actively growing *Vibrio* can have 100× the biomass of *Pelagibacter*, which, at ~10^5^ cells per ml, is typically the most abundant heterotrophic member of bacterial assemblages in the ocean (Yooseph et al., 2010). Second, new time-series analysis shows that vibrios are capable of blooms in the water column during which they can even become the predominant members of the total bacterial assemblage (Gilbert et al., 2012). These blooms had been missed previously because they are of relatively short duration, yet they confirm that vibrios, which are capable of very rapid growth in laboratory media, can reach high doubling rates in the environment. Finally, vibrios might be disproportionately subject to predation by protozoa and viruses (Worden et al., 2006; Suttle, 2007), likely due to their comparatively large size. For example, cells were found in one study to measure more than three times the community average in volume, and, along with other similarly large genera, suffered especially high grazing mortality (Beardsley et al., 2003). Taken together, these considerations suggest that vibrios should be re-evaluated for their role in biogeochemical processes in the ocean since they have disproportionately high biomass that is subject to high turnover by rapid growth in concert with high predation.

The purpose of this review is to provide an overview of known environmental factors and ecological associations affecting *Vibrio* abundance and dynamics. We note that although we look at the dynamics of potentially pathogenic species, we purposefully exclude data on pathogenesis itself since this is outside the scope of this review. We first focus on total *Vibrio* (i.e., the assessment of occurrence of members of the genus or family), which have often been measured as a proxy for potential pathogen occurrence, asking whether they can be treated as an environmentally cohesive unit. To what extent do total vibrios correlate to specific environmental variables, and do these measures have predictive power for individual species? To address this question, we present meta-analyses of the dynamics of *V. cholerae*, *V. parahaemolyticus*, and *V. vulnificus*, three species harboring genotypes potentially pathogenic to humans. The limitation to these three is necessary since public health interests have driven much of the research so that the literature is highly biased toward human pathogens. In this context, a further important question is to what extent easily measurable bulk parameters, such as temperature, salinity, nutrients, dissolved oxygen and/or chlorophyll a are good correlates for total vibrios or specific species, allowing easy and cost-effective risk assessment.

However, because our meta-analysis suggests poor or inconsistent performance of most bulk parameters, we researched alternative, frequently finer-scale environmental variables. These include associations with different animals, plants and algae, as well as organic polymers, which may occur as suspended particulate matter in the water column and provide resources for attached bacteria. Although such attached lifestyles are common for vibrios, recent research also suggests that many species can occur free-living at least part of the time and be engaged in relative short-lived blooms.

Finally, we summarize recent research aimed at defining habitat characteristics and phylogenetic bounds of ecologically cohesive populations among co-existing vibrios, using the water column and macroinvertebrates as examples of adaptive landscapes. This research demonstrates that such populations, which may or may not correspond to named (taxonomic) species, represent eco-evolutionary units that allow testing of hypotheses of how populations are structured by environmental selection and gene flow.

## Environmental correlates of *Vibrio* presence and abundance

To better understand under what conditions vibrios occur and proliferate, most studies have investigated environmental variables that can be measured from bulk seawater such as temperature, salinity, dissolved oxygen, nitrogen, phosphorus, and chlorophyll a concentrations. These are attractive since they are easily measured and many are observable remotely by buoy or satellite (e.g., Lobitz et al., 2000) so that potential for presence of pathogenic vibrios might be easily assessed. In addition, several studies have extended measurements to more complex physicochemical and biotic variables, including dissolved organic carbon (DOC) and zoo- and phyto-plankton taxa.

In the following, we first ask how informative these variables are by conducting a meta-analysis to compare correlations across studies, for both total *Vibrio* as well as the potential pathogens *V. cholerae, V. parahaemolyticus*, and *V. vulnificus*, and, second, determine if the genus and species levels exhibit similar patterns. To determine the potential impact of environmental variables, we looked at how strong their correlations are by comparing coefficient of determination values, *R*^2^, reported in the literature. A goodness of fit parameter, *R*^2^ varies from 0 (no explanation of variance in the dependent variable) to 1 (perfect explanation), giving us a means of assessing, for example, whether temperature better predicts abundance of total *Vibrio*, than salinity does. Studies included have regression analyses with associated *R*^2^-values, or Spearman or Pearson correlations, whose rho values were squared to obtain *R*^2^. Additionally, we compare how their abundances trend along gradients in two particularly well-studied variables, salinity and temperature.

### Total *Vibrio*

**Figure 1:**
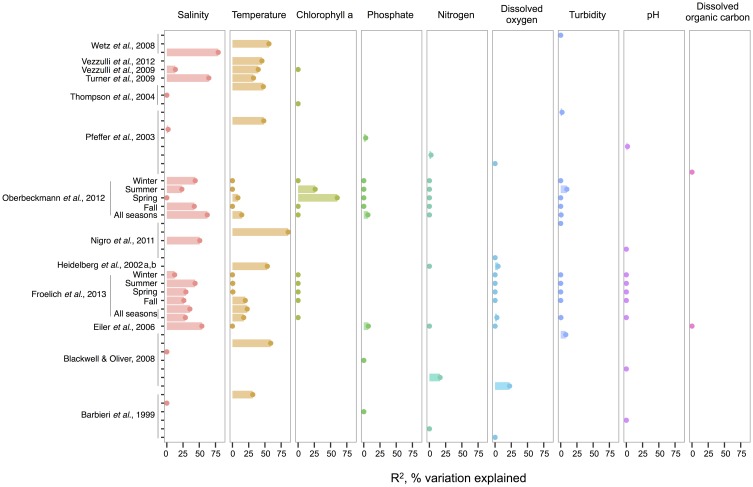
**An overview of regression analyses indicate that temperature and salinity explain most variation in bulk-water total *Vibrio* abundance**. The *R*^2^, or pseudo-*R*^2^, values associated with regression analyses are shown for selected environmental variables that are well-represented across studies. An individual study may perform multiple analyses because variables are considered for correlation independently (for ex. Wetz et al., 2008); because datasets are split (e.g., between seasons in Oberbeckmann et al., 2012); or because different sets of variables are considered sequentially (e.g., two variables versus six variables in the two All Seasons models from Froelich et al., 2013). Dots indicate bar heights, and where a dot occurs without a bar, *R*^2^ was non-significant (i.e., *R*^2^ = 0). Variables may have been log or exponentially transformed in references.

When correlations across studies are compared, we see that the strongest environmental correlates to total *Vibrio* are temperature and salinity. These two variables most often explain the greatest amount of variance in total *Vibrio* abundance in the water column (Figure 1), whereas consideration of additional variables often makes only marginal improvements (e.g., in Heidelberg et al., 2002a,b; Oberbeckmann et al., 2012; Froelich et al., 2013). However, a minority of analyses has found temperature and salinity to be non-significant toward explaining *Vibrio* abundance. This inconsistency might be a result of the ranges considered; for instance, temperature may be found non-significant due to a narrow range observed, such that *Vibrio* abundance varies little. In fact, evidence supports this hypothesis; the correlation strength of temperature to vibrios varies by season (Oberbeckmann et al., 2012; Froelich et al., 2013), suggesting the magnitude of the correlation may depend on the temperature range examined. For instance, Oberbeckmann et al. (2012) and Froelich et al. (2013) both observed the highest correlation of temperature and *Vibrio* during the seasons with the broadest temperature ranges, spring, and fall, respectively. Additionally, it is possible that at lower temperatures vibrios exhibit less variation in abundance; two studies assessing total vibrios in the cooler waters of the Baltic Sea and North Sea found non-significant correlations (Eiler et al., 2006; Oberbeckmann et al., 2012).

Compared to salinity and temperature, other environmental measures usually explain less variance in total *Vibrio*. Dissolved oxygen has had little explanatory power; for instance, in Figure 1, its largest *R*^2^ was less than half that of temperature in the same analysis (Blackwell and Oliver, 2008). The same is true for nitrogen, whose highest *R*^2^ was still less than temperature's (Blackwell and Oliver, 2008). In the environments examined, phosphate, pH, and turbidity explain little variance, and DOC explains none at all, albeit the number of studies used for DOC in this meta-analysis is limited. Of interest, though not depicted, potential host organisms, copepods, decapods, and cyanobacteria, have been found to explain relatively little variance in total vibrios when considered in a model that already incorporates temperature (Turner et al., 2009; Vezzulli et al., 2009), and similarly for dinoflagellates when salinity is first considered (Eiler et al., 2006). Turner et al. (2009) did observe that diatoms explained more variance than temperature. While this might imply a physical association, the correlation was negative, suggesting that total *Vibrio*, at least as a whole, do not associate with diatoms.

Chlorophyll a, on the other hand, has had noted importance in two datasets: the spring and summer of the study by Oberbeckmann et al. (2012), with *R*^2^-values of 60 and 26%, respectively. These were in fact higher than correlations to temperature or salinity in these seasons. Perhaps during this period, as temperature warms, growth conditions favor phytoplankton blooms that impact *Vibrio* abundance (Oberbeckmann et al., 2012). However, Froelich et al. (2013) did not make these same observations in their seasonal datasets. This inconsistency may be a product of the fact that different *Vibrio* species likely affiliate with or feed on exudates of specific algal taxa only, rather than algae in general, a subject further discussed in the section The Evidence for a Planktonic, Free-Living Lifestyle.

Given the frequent strength of temperature and salinity as correlates, we asked, how do total vibrios distribute with respect to these variables when their combined effect is considered? A few studies have modeled the bivariate relationship, finding that total *Vibrio* abundance increases as temperature and salinity increase (Hsieh et al., 2008; Turner et al., 2009; Froelich et al., 2013). The ranges investigated were also broad, lending confidence that these results are general; for example, Hsieh et al. (2008) modeled from 2.5 to 32.5°C and 0 to 27 ppt, respectively.

### *V. cholerae, V. parahaemolyticus*, and *V. vulnificus*

We compare environmental correlates and trends noted in total *Vibrio* to three species that have been well-sampled across locales: *V. cholerae, V. parahaemolyticus*, and *V. vulnificus*. While it would also be interesting to consider species beyond potential pathogens, their environmental data is much more limited.

**Figure 2:**
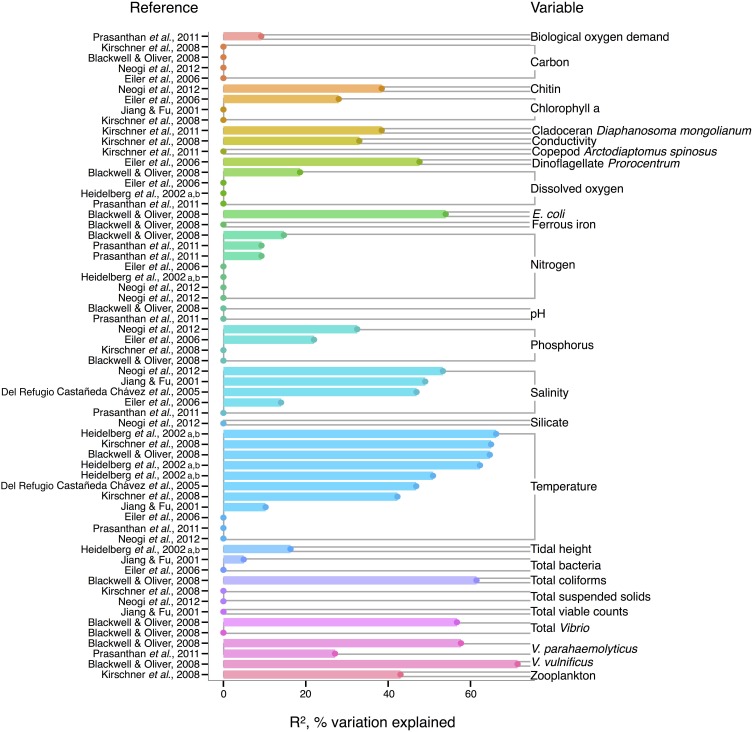
**Variation in *V. cholerae* abundance or percent positive samples is best explained by temperature, other organisms, and salinity**. *R*^2^, or pseudo-*R*^2^, values from analyses across studies are depicted grouped by variable, and then in rank order, with their associated reference. A reference may conduct multiple analyses for a given variable (e.g., on subsets of data or considering different variables combinations for data regression). Dots indicate bar heights, and where a dot occurs without a bar, *R*^2^ was non-significant (i.e., *R*^2^ = 0).

In *V. cholerae*, we see an interesting shift from total *Vibrio* in the strength of correlating environmental variables: some biotic variables are as strong or, in fact, stronger than temperature or salinity (Figure 2). Total *Vibrio*, congenerics *V. vulnificus* and *V. parahaemolyticus*, as well as a dinoflagellate genus (*Prorocentrum*) and cladoceran species (*Diaphanosoma mongolianum*) have all significantly correlated to *V. cholerae* abundance (Eiler et al., 2006; Blackwell and Oliver, 2008; Kirschner et al., 2011; Prasanthan et al., 2011). Moreover, *V. parahaemolyticus* abundance has explained more *V. cholerae* abundance variance than nitrogen, temperature, or salinity in (Prasanthan et al., 2011), and dinoflagellate abundance has explained more variance than phosphorus, salinity, or temperature (Eiler et al., 2006). While correlations to plankton may represent direct associations, such high correlation of vibrios to each other is likely not indicative of causal interactions, but rather stems from overlap in environmental ranges and/or habitats (Blackwell and Oliver, 2008). *E. coli* and total coliforms have also correlated to *V. cholerae* abundance, though both groups may simply be responding to anthropogenic nutrient influxes favoring growth of heterotrophs (Blackwell and Oliver, 2008).

Long thought to be a reservoir of toxigenic *V. cholerae*, zooplankton, and particularly copepods, are hypothesized to correlate to *V. cholerae* abundance. Surprisingly, however, when de Magny et al. (2011) examined several zooplankton genera and species, including copepods *Cyclops* and *Diaptomus*, they did not find significant correlations to any zooplankter except the rotifer *Brachionus angularis* (not depicted in Figure 2, because Monte Carlo analysis did not yield *R*^2^-values). While the association between *V. cholerae* O1/O139 and the copepod *Acartia tonsa* has also been studied (Huq et al., 2005; Lizárraga-Partida et al., 2009), quantitatively significant correlation in the environment has remained elusive. For instance, Lizárraga-Partida et al. (2009) demonstrated only a qualitative link between *V. cholerae* O1 presence coincident with an increase in *A. tonsa*, even though laboratory studies have shown ready attachment (e.g., Huq et al., 1984; Rawlings et al., 2007).

*V. cholerae* has also been hypothesized to correlate with chlorophyll a, a potential proxy of algal and zooplankton growth, and/or a eutrophic environment conducive to heterotroph growth, but chlorophyll a's general predictive value is unclear. While significant in Eiler et al. (2006), other studies have observed no correlation of chlorophyll a to *V. cholerae* abundance (Jiang and Fu, 2001; Kirschner et al., 2008; Mishra et al., 2012). Yet *V. cholerae* growth has been observed experimentally to depend on DOC, which could relate to phytoplankton abundance and thus chlorophyll a (Eiler et al., 2007). In microcosm experiments, Eiler et al. (2007) demonstrated that adding 2.1 mg carbon L^−1^ of cyanobacterial-derived dissolved organic matter influenced bacterial growth more than a 12–25°C change in temperature. The inconsistency of chlorophyll a, and, incidentally, bulk DOC (which showed no significant correlation) (Eiler et al., 2006; Blackwell and Oliver, 2008; Kirschner et al., 2008; Neogi et al., 2012) as correlates might be due to the quality of exudates; its composition of refractory humic substances (Kirschner et al., 2008) or derivation from different algal species, differentially stimulating *V. cholerae* growth [(Worden et al., 2006), see also section The Evidence for a Planktonic, Free-Living Lifestyle]. Interestingly, the lack of clear support for chlorophyll a's influence on *V. cholerae* environmental abundance is in contrast to the fact that chlorophyll a can correlate with cholera *disease* incidence (de Magny et al., 2008), and has been used in predictive models for cholera in Bangladesh (Bertuzzo et al., 2012; Jutla et al., 2013).

**Figure 3:**
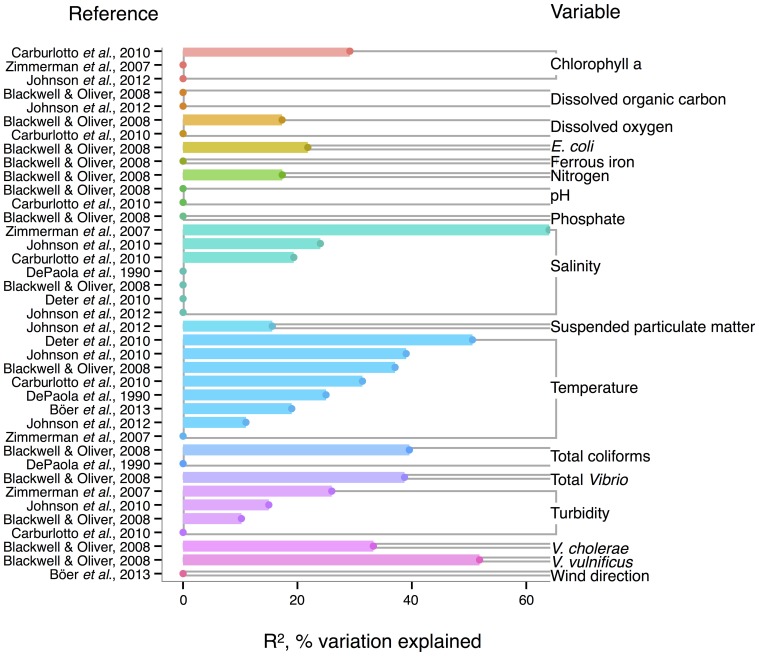
**Variation in *V. parahaemolyticus* abundance or percent positive samples is best explained by temperature and other organisms**. *R*^2^, or pseudo-*R*^2^, values from analyses across studies are depicted grouped by variable, and then in rank order, with their associated reference. A reference may conduct multiple analyses for a given variable (e.g., on subsets of data or considering different variables combinations for data regression). Dots indicate bar heights, and where a dot occurs without a bar, *R*^2^ was non-significant (i.e., *R*^2^ = 0).

Like *V. cholerae*, *V. parahaemolyticus* abundance in water samples is also strongly correlated to temperature, and was found significant in all but one analysis reviewed here (DePaola et al., 1990; Zimmerman et al., 2007; Blackwell and Oliver, 2008; Caburlotto et al., 2010; Deter et al., 2010; Johnson et al., 2010, 2012; Böer et al., 2013), with maximal *R*^2^ = 50.6% (Deter et al., 2010) (Figure 3). Blackwell and Oliver (2008) found that *V. parahaemolyticus* correlates both to total *Vibrio* and congenerics, as well as coliforms and *E. coli*. These variables were only considered in a single study, however, so it is not known if the relationships hold across different sampling locations. The significance of salinity is variable for *V. parahaemolyticus* with only three of seven studies having non-zero *R*^2^-values (Figure 3) (Zimmerman et al., 2007; Caburlotto et al., 2010; Johnson et al., 2010), but this may be due to *V. parahaemolyticus* colonizing a large salinity range, as detailed below (Figure 6).

Correlation to environmental variables has also frequently been studied for *V. parahaemolyticus* occurring in sediment and shellfish, though trends remain unclear. In sediment, considered a potential reservoir (Vezzulli et al., 2009), individual regressions of *V. parahaemolyticus* abundance to temperature, salinity, and total organic carbon have yielded moderate *R*^2^-values, at times above 30% (Blackwell and Oliver, 2008; Deter et al., 2010; Johnson et al., 2012; Böer et al., 2013). However, some studies have found salinity or temperature to be a non-significant explanatory variable (Blackwell and Oliver, 2008; Deter et al., 2010; Johnson et al., 2010).

In shellfish, a common vehicle of virulent vibrios to humans, the incidence of temperature and salinity as correlates to *V. parahaemolyticus* is also inconsistent. Salinity has been found explanatory in some studies, with *R*^2^ as high as 42% (DePaola et al., 2003; Johnson et al., 2010, 2012) and non-significant in others (Deepanjali et al., 2005; Deter et al., 2010; Sobrinho et al., 2010). Temperature can explain moderate amounts of variance in *V. parahaemolyticus* abundance (DePaola et al., 1990, 2003; Cook et al., 2002; Johnson et al., 2010, 2012; Sobrinho et al., 2010), with significant *R*^2^ as high as 44% (Cook et al., 2002), though other studies have found little or no correlation (Deepanjali et al., 2005; Duan and Su, 2005; Deter et al., 2010). The absence of correlation is surprising, given that temperature's effect is amplified by influencing shellfish's ability to concentrate *V. parahaemolyticus* from surrounding water. Oysters can enrich *V. parahaemolyticus* over 100-fold (DePaola et al., 1990; Shen et al., 2009), and the magnitude of concentration is temperature-dependent, with effects greatest at 32°C and less, but still evident, in cooler waters (Shen et al., 2009).

**Figure 4:**
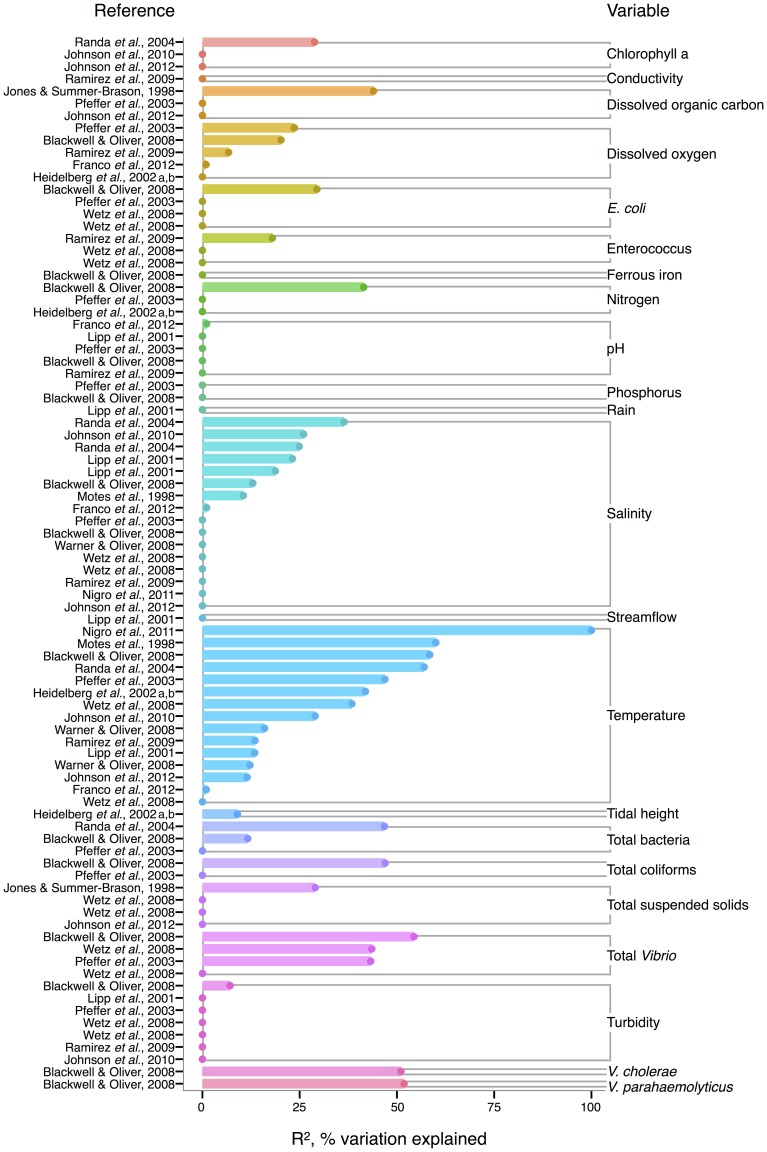
**Variation in *V. vulnificus* abundance or percent positive samples is best explained by temperature, and other organisms, including *Vibrio***. *R*^2^, or pseudo-*R*^2^, values from analyses across studies are depicted grouped by variable, and then in rank order, with their associated reference. A reference may conduct multiple analyses for a given variable (e.g., on subsets of data or considering different variables combinations for data regression). Dots indicate bar heights, and where a dot occurs without a bar, *R*^2^ was non-significant (i.e., *R*^2^ = 0).

For *V. vulnificus* isolated from the water column, temperature is the strongest correlate among measured environmental variables, and often explains more variance in *V. vulnificus* than for other species or total *Vibrio*; several analyses found temperature explained over 50% of the variance in *V. vulnificus* sampled from water (Motes et al., 1998; Randa et al., 2004; Blackwell and Oliver, 2008; Nigro et al., 2011) (Figure 4). Moreover, temperature has been a stronger correlate than chlorophyll a (Randa et al., 2004; Johnson et al., 2010, 2012), dissolved oxygen (Pfeffer et al., 2003; Blackwell and Oliver, 2008; Ramirez et al., 2009), and nitrogen (Pfeffer et al., 2003; Blackwell and Oliver, 2008). While DOC is an inconsistent correlate, it has been more explanatory than temperature in at least one study (Jones and Summer-Brason, 1998). The variable pH, however, is not a significant correlate (Lipp et al., 2001; Pfeffer et al., 2003; Blackwell and Oliver, 2008; Ramirez et al., 2009; Franco et al., 2012), nor is phosphorus (Pfeffer et al., 2003; Blackwell and Oliver, 2008). Turbidity has been found non-significant in several studies (Lipp et al., 2001; Pfeffer et al., 2003; Wetz et al., 2008; Ramirez et al., 2009), or not as explanatory as temperature (Blackwell and Oliver, 2008). While salinity, when significant, has generally been less informative than temperature (Motes et al., 1998; Randa et al., 2004; Warner and Oliver, 2008; Johnson et al., 2010), it has, in one analysis, been more (Lipp et al., 2001).

Biotic correlates have also been identified for *V. vulnificus*. Total bacteria (Pfeffer et al., 2003; Randa et al., 2004; Blackwell and Oliver, 2008), enteroccous (Wetz et al., 2008; Ramirez et al., 2009), coliforms (Pfeffer et al., 2003; Blackwell and Oliver, 2008) and *E. coli* (Pfeffer et al., 2003; Blackwell and Oliver, 2008; Wetz et al., 2008) have been studied only sporadically, but their correlation strength to *V. vulnificus* has usually been less than temperature's; one exception, however, is enterococcus in (Ramirez et al., 2009), potentially indicative of a surge in nutrients overtaking temperature's effect on growth. Interestingly, total *Vibrio* have explained substantial variance (*R*^2^ = 43–54%) in *V. vulnificus* in more instances than for other *Vibrio* species (Pfeffer et al., 2003; Blackwell and Oliver, 2008; Wetz et al., 2008), suggesting they are responding similarly to their environments under the conditions studied. However, instances do occur where total *Vibrio* and *V. vulnificus* do not correlate (Høi et al., 1998; Wetz et al., 2008), underscoring that a species is not a constant component of a genus, and may respond to environmental conditions independently.

**Figure 5:**
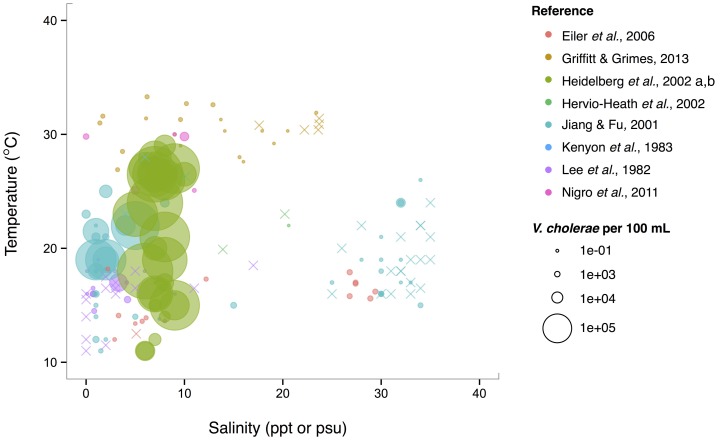
***V. cholerae* favors lower salinity and occupies a broad temperature range**. *V. cholerae* concentrations, i.e., MPN-estimated CFU or molecular marker gene copies per 100 mL, reported in different studies are plotted against the temperature (°C) and salinity values (ppt or psu) at which they were found. All studies report *V. cholerae*, including O1/O139 and non-O1/non-O139, except for Heidelberg et al. (2002a,b); DeLoney-Marino et al. (2003), whose genetic marker detected *V. cholerae/V. mimicus*. Circle (°) sizes correspond to concentrations, but note the breaks are scaled for clearer visualization, and not linearly. (×) indicates no *V. cholerae* found in that sample.

Isolations of the three potentially pathogenic species across salinity and temperature gradients were also looked at, and found to exhibit different patterns. *V. cholerae* has a wide temperature range (~10–30°C) in brackish water (1–10 ppt), and generally decreases with increasing salinity over the entire range examined (0–40 ppt) (Figure 5). Observed *V. cholerae* abundance is greatest around 20°C and 0–10 ppt, on the order of 10^3^ cells per mL. At less-favorable, higher salinities, *V. cholerae* has been found around this temperature, though in much lower abundances (on the order of 1 cell per mL). Interestingly, *V. cholerae*'s realized niche is much smaller than its fundamental one, as it has maximal temperature and salinity tolerances around 38°C and 75 ppt (Materna et al., 2012), suggesting other controls on its abundance in the environment.

**Figure 6:**
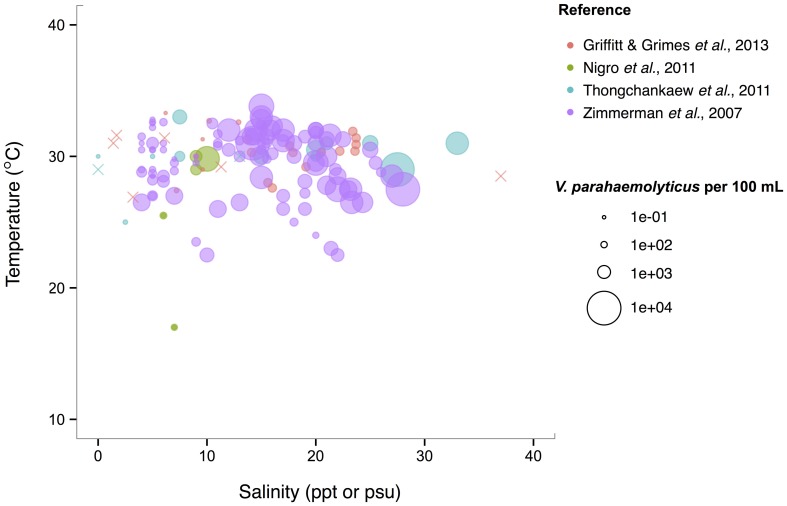
***V. parahaemolyticus* favors high temperatures but is relatively unconstrained by salinity**. Concentrations, i.e., MPN-estimated CFU or molecular marker gene copies per 100 mL, reported in different studies are plotted against the temperature (°C) and salinity values (ppt or psu) at which they were found in bulk water samples. Circle (°) correspond to concentrations, but note the breaks are scaled for clearer visualization, and not linearly. (×) indicates no *V. parahaemolyticus* found in that sample.

*V. parahaemolyticus* contrasts *V. cholerae* by having a more constant abundance that is broadly spread out over salinities of 3–35 ppt in a narrow, much warmer temperature range, centered roughly around 29°C. (Figure 6). Consistent with this finding, it has been noted that this species prefers warmer waters (>20°C) (Martinez-Urtaza et al., 2012), and has been observed to grow best at 25°C *in vitro* (Nishina et al., 2004). However, isolations from shellfish can exhibit different trends from those observed in the water column; Martinez-Urtaza et al. (2008) detected *V. parahaemolyticus* in mussels gathered in much cooler, 15°C water, consistent with the potential for shellfish to concentrate *V. parahaemolyticus*.

A previous literature-based analysis showed *V. vulnificus* to have a more complicated relationship to temperature and salinity than either *V. cholerae* or *V. parahaemolyticus*. It has a narrow temperature range at higher salinities (>10 ppt) while at low salinities (between 5 and 10 ppt) its temperature range more than doubles—from 22–30°C to 10–32°C (Randa et al., 2004). This suggests that, in temperate climates, this species is found year-round in estuarine, low salinity environments but can expand into full strength seawater during warmer months. In the tropics, this species should be endemic to the ocean.

### Conclusions from meta-analysis

From this meta-analysis, we find, first, that temperature and salinity often explain more variance than any other bulk water parameter, like phosphate, nitrogen, pH, or DOC. Yet some of the difficulty in making general statements regarding the relationship of vibrios to individual environmental variables likely stems from the fact that their strength can depend on the ranges examined, e.g., as for temperature, or in quality of the variable, such as DOC, which will encompass carbon derived from different sources that may impact *Vibrio* growth differentially. Second, we observe that trends that apply to the whole genus *Vibrio* do not necessarily reflect those of individual species. Total vibrios and the well-studied potential pathogens *V. cholerae*, *V. parahaemolyticus*, and *V. vulnificus* correlate with shared and distinct environmental variables. For *V. parahaemolyticus* and *V. vulnificus*, temperature often explains more variance than does salinity in the same analysis, and for *V. cholerae*, diverse biotic variables, including specific phyto- and zooplankton taxa, can be stronger correlates than abiotic variables. Unfortunately, biotic variables, particularly individual plankton taxa, have rarely been studied in more than one instance, making these observations difficult to generalize. But the correlations reviewed above hint that there may be ecological relationships between *Vibrio* and plankton that merit deeper investigation.

Across salinity and temperature gradients, the pattern also differs between total *Vibrio* and individual species, and species' patterns differ from each other. Indeed, differences may occur even within taxonomic species; *V. parahaemolyticus* pathogenic genotypes have been observed to be a variable fraction of total *V. parahaemolyticus* (Zimmerman et al., 2007). For example, at their Alabama site, total *V. parahaemolyticus*—detected via thermolabile hemolysin marker (tlh)—remained at a more constant concentration of between 1 and 10 cells per mL, while toxigenic genotypes—thermolabile hemolysin+ and thermostable direct hemolysin+ cells—fluctuated in a much wider range: between 0.0001 and 10 cells per mL. This result argues against using the total species to infer the potential pathogens. Taken together with the results from the meta-analysis, these findings suggest that finer-scale sampling—of both the environmental parameters and the *Vibrio* population of interest—is necessary to link ecological parameters to cellular abundances.

## Associations with complex and particulate marine growth substrates

The previous sections demonstrate that, with the exception of temperature and salinity, parameters measured in bulk seawater have shown limited power in explaining the environmental dynamics of *Vibrio* species. This may, in part, be due to the narrow focus on only a few (potentially) pathogenic species, and frequently limited comparability of measured parameters across studies. It is also likely, however, that bulk measurements, such as dissolved oxygen, nitrogen and phosphate concentration in seawater, only poorly capture the ecological parameters that *Vibrio* populations are associated with or respond to. Vibrios are often presumed to primarily attach to biological surfaces, yet may also subsist on dissolved resources of biological origin while free-living. Taking these resource associations into account, their environmental dynamics may be somewhat decoupled from parameters measurable in bulk seawater, and may depend more on the concentration and properties of relevant solid or dissolved resources. We review in the following sections the ample evidence for surface-associated niches, as well as more recent evidence for environmental dynamics including free-living states and formation of blooms.

From the perspective of bacteria attaching to surfaces, these are either metabolically inert or can be degraded as a source of growth substrates. Vibrios have the ability to attach to and degrade a considerable number of polymeric substrates (Johnson, 2013), suggesting that specific association with surfaces is an important growth strategy. For example, nearly all vibrios can metabolize the abundant biopolymer chitin (present in both crustacean and diatom shells in the marine environment) (Hunt et al., 2008a; Grimes et al., 2009), and various representatives can metabolize an array of plant/algal polysaccharides: agar, alginate, fucoidan, mannan, cellulose, pectin, and laminarin (Goecke et al., 2010). In addition, vibrios may metabolize plastic wastes, as suggested by a recent study documenting that vibrios make up the majority of bacteria attached to plastic wastes floating in the ocean, with electron microscopy showing individual cells residing at the bottom of pits (Zettler et al., 2013). Although this suggests that these plastics, which had been thought to be largely biologically inert, could be degraded by vibrios, such activity remains to be confirmed.

Evidence is also accumulating that vibrios may play a role in oil spill degradation: *Vibrio* representatives can metabolize oil-derived compounds (West et al., 1984; Moxley and Schmidt, 2010), and have been found to comprise a sizable fraction of oil-associated microbial communities from the Deepwater Horizon spill, both from sea-surface samples (>31% in the molecular study of Hamdan and Fulmer, 2011) and salt-marsh plants contaminated with oil mousse (57% in the study of Liu and Liu, 2013). While a clear positive effect of crude oil on *Vibrio* growth has yet to be demonstrated *in vitro*, it appears that many vibrios can at least persist in the presence of oil (Stephens et al., 2013). *Vibrio* representatives furthermore show resistance to inhibition by the oil dispersant Corexit (Hamdan and Fulmer, 2011), which was widely used following the Deepwater Horizon spill; this resistance may additionally support an ability to persist after oil spills.

Most associations with specific surfaces have, however, been described for plants, algae, and animals, and the following section explores these organisms as potential biological niches for vibrios.

## Biological niches for *Vibrio*

*Vibrio* have been detected on a plethora of aquatic biological surfaces, but which of these associations represent more than transient, incidental attachments? In the following sections we consider which aquatic plants (Table 1) and animals (Table 2) may represent sustained *Vibrio* niches, on the basis of (1) numerical enrichment compared to the surrounding medium, and (2) knowledge of biological mechanisms, e.g., availability of nutrition and shelter, potentially supporting an association. In doing so, we also draw attention to the need for more quantitative and mechanistic approaches to understanding the ecological associations that allow vibrios to flourish—approaches that could underpin more powerful predictions of *Vibrio* dynamics arising from these diverse associations. We note also that many of the following observations are limited to *V. cholerae* because of its prominence as a pathogen, but the same niches may be available to other vibrios with similar biological activities.

**Table 1:** **Plant and algae hosts for vibrio, as demonstrated by numerical enrichment and biological mechanisms supporting association**.

**Host**	**References, study site**	**Associated *Vibrios***	**Enumeration method**	**Enrichment, survival advantage**	**Host site, mechanism of association**
**PLANTS, FRESHWATER**					
*Eichhornia crassipes* (water hyacinth)	Spira et al., 1981: Bangladesh, freshwater bodies	*V. cholerae* O1 El Tor	Culture	*In situ* enrichment: 84% incidence on plants, 16% in water only. *In vitro* survival advantage: enriched by 10^2^–10^3^ compared to surrounding water	Possible preference for root exudate
*Lemna minor* (duckweed)	Islam et al., 1990b: *in vitro*	*V. cholerae* O1: one clinical strain, one environmental (from Australian river water)	Culture	*In vitro* survival advantage: >27 days survival of attached cells, vs. 15–21 days for cells in surrounding water	Whole plant; mechanism untested
**PLANTS, ESTUARINE**					
*Spartina alterniflora, Spartina patens* (marsh grass)	Bagwell et al., 1998 Lovell et al., 2008; Gamble et al., 2010: South Carolina estuary, USA	Spp. including *V. alginolyticus, anguillarum, diazotrophicus, parahaemolyticus*	Culture; molecular	*In situ* enrichment: >50% of culturable diazotrophs; molecular evidence (Gamble et al., 2010) demonstrates stable abundance across seasons	Root association; anaerobic diazotrophy
*Juncus roemarianus* (marsh grass)	Larocque et al., 2004: South Carolina estuary, USA	*Vibrionaceae*	Culture	*In situ* enrichment: >50% of culturable diazotrophs	Root association; anaerobic diazotrophy
*Salicornia viginica* (marsh herb)	Bergholz et al., 2001; Criminger et al., 2007: South Carolina estuary, USA	*Vibrionaceae*	Culture	*In situ* enrichment: >50% of culturable diazotrophs	Root association; anaerobic diazotrophy
**MICROALGAE AND FILAMENTOUS CYANOBACTERIA, FRESHWATER**					
*Rhizoclonium fontanum* (filamentous green alga)	Islam et al., 1989: *in vitro*	*V. cholera*e O1 strains from Australian and Bangladeshi surface water; O1 Bangladeshi clinical isolates	Culture	*In vitro* survival advantage: 21 days survival of attached cells, compared to 3 days in surrounding water and in no-algae control	Mechanism untested
*Anabaena variabilis*	Islam et al., 1990b, 2002, 2006; Mizanur et al., 2002: *in vitro*	*V. cholerae* O1 Bangladeshi environmental isolates	Culture	*In vitro* survival advantage: up to 5 days survival of attached cells; >6 survival in associated water. Persist as VBNC inside algal sheath up to 15 months	Mucilaginous sheath, with possible preference for heterocysts. Possible mechanism: benefiting from algal exudate while relieving oxygen inhibition of N_2_ fixation and contributing CO_2_. Demonstrated mechanisms: chemotaxis to host mucus components; mucinase dependence of both chemotaxis and survival with host
**MACROALGAE, MARINE**					
*Brown algae*					
*Ascophyllum nodosum*	Chan and McManus, 1969: Canada	*Vibrio* spp.	Culture	*In situ* enrichment: Dominant culturable bacteria; enriched by 10^2^–10^4^ compared to water column	Algal polysaccharide metabolism
*Laminaria* spp.	Laycock, 1974: Nova Scotia, Canada; Wang et al., 2009	Spp. incl. *V. tasmaniensis*	Culture	*In situ* enrichment: Dominant culturable bacteria	Algal polysaccharide metabolism; laminaranolytic activity in particular demonstrated
*Red algae*					
*Hypnea* spp.	Lakshmanaperumalsamy and Purushothaman, 1982: tropical estuary, Africa	*Vibrio* spp.	Culture	*In situ* enrichment: Dominant culturable bacteria	Algal polysaccharide metabolism
*Polysiphonia lanosa*	Chan and McManus, 1969: Canada. Islam et al., 1988: *in vitro*.; Wang et al., 2009	*Vibrio* spp., incl. *V. tasmaniensis, splendidus*; *in vitro* experiments with *V. cholerae* O1	Culture	*In situ* enrichment: Dominant culturable bacteria; enriched by 10^2^–10^4^ compared to water column. *In vitro* survival advantage demonstrated	Algal polysaccharide metabolism
*Porphyra yezoensis*	Duan et al., 1995: China	*Vibrio* spp.	Culture, scanning electron microscopy	*In situ* enrichment: Dominant microscopically identifiable and culturable bacteria	Algal polysaccharide metabolism
*Green algae*					
*Chaetomorpha* spp.	Lakshmanaperumalsamy and Purushothaman, 1982: tropical estuary, Africa	*Vibrio* spp.	Culture	*In situ* enrichment: Dominant culturable bacteria	Algal polysaccharide metabolism
*Enteromorpha intestinalis, linza*	Lakshmanaperumalsamy and Purushothaman, 1982: tropical estuary, Africa Islam et al., 1988: *in vitro*	*Vibrio* spp.; *in vitro* experiments with *V. cholerae* O1	Culture	*In situ* enrichment: Dominant culturable bacteria. *In vitro* survival advantage demonstrated	Algal polysaccharide metabolism
*Ulva lactuca, pertusa*	Islam et al., 1988: *in vitro*; Duan et al., 1995: China; Nakanishi et al., 1996; Patel et al., 2003; Tait et al., 2005	*Vibrio* spp.; *in vitro* experiments with *V. cholerae* O1	Culture, scanning electron microscopy	*In situ* enrichment: Dominant microscopically identifiable and culturable bacteria. *In vitro* survival advantage demonstrated	Algal polysaccharide metabolism; modulation of host processes: developmental morphogenic effects, spore germination stimulation

**Table 2:** **Animal hosts for vibrio, as demonstrated by numerical enrichment and biological mechanisms supporting association**.

**Host**	**References, study site**	**Associated vibrios**	**Enumeration method**	**Enrichment, survival advantage**	**Host site, mechanism of association**
**INVERTEBRATES**					
*Freshwater*					
*Acanthamoeba* protozoa	Abd et al., 2005, 2007, 2010; Sandström et al., 2010: *in vitro*	*V. cholerae* O1, O139; *V. mimicus*	Culture, microscopy	*In vitro* survival advantage: replicate intracellularly >14 days	Cytoplasm, cysts; protected from antibiotics and predation
Chironomid midge egg masses	Broza and Halpern, 2001; Halpern et al., 2003, 2008: *in vitro*	*V. cholerae* isolates from Israeli rivers and waste-stabilization ponds	Culture	*In vitro* survival advantage: 10^3^ greater cell counts compared to growth in medium alone	Gelatinous egg matrix; can use gelatinous material as sole carbon source, degrading via secreted hemagglutinin/protease
Zooplankton: cladoceran *Diaphanosoma mongolianum*, from alkaline lake, Germany	Kirschner et al., 2011: *in vitro*	*V. cholerae* non-O1/non-O139 isolate from alkaline lake, Germany	Fluorescence *in situ* hybridization	*In vitro* survival advantage, but not enrichment: up to 6-fold increase in growth rate of cells in surrounding medium; 10^5^–10^7^ cells attached compared to 10^6^–10^7^ cells in surrounding medium	Probable use of host exudates
*Estuarine and marine*					
Zooplankton: Estuarine copepods, espp. *Acartia* and *Eurytemora*	Simidu et al., 1971: Japan; Sochard et al., 1979: Gulf of Mexico; Huq et al., 1983, 1984: *in vitro*; Colwell, 1996: *in vitro*; Mueller et al., 2007: *in vitro*; Preheim et al., 2011a: Massachusetts estuary, USA	*Vibrio* spp., espp. *V. cholerae*	Culture	*In situ* and *in vitro* enrichment shown in some cases, with up to 10^5^ cells per host. Can dominate culturable surface- and gut-attached communities	Possible preference for oral region and egg sac, due to proximity to host exudates; preference for live versus dead hosts unclear
Corals, incl. *Acropora hyacinthus*, *Oculina patagonica*, *Mussimilia hispida*, *Stylophora pistillata*	Koren and Rosenberg, 2006: Israel; Kvennefors et al., 2010: Great Barrier Reef; Chimetto et al., 2008; Sharon and Rosenberg, 2008; Koenig et al., 2011; Krediet et al., 2013	Spp. incl. *V. alginolyticus, harveyi, splendidus*	Culture, molecular	*In situ* enrichment: can dominate mucus community, according to both culturing and molecular methods; can dominate culturable diazotrophs (found for *Mussimilia hispida*)	Mucus. Metabolize mucus; diazotrophs likely contribute nitrogen to hosts; may adapt to host antimicrobials via antibiotic-resistance gene acquisition; can inhibit pathogen colonization
Shellfish: blue crabs, *Callinectes sapidus*	Davis and Sizemore, 1982: Texas, USA	Spp. incl. *V. cholerae, vulnificus, parahaemolyticus*	Culture	*In situ* enrichment: Dominant culturable bacteria in hemolymph	Hemolymph; mechanism untested
Shellfish: oysters	Murphree and Tamplin, 1995; Froelich and Oliver, 2013	Spp. incl. *V. cholerae, parahaemolyticus, vulnificus*	Culture	*In situ* enrichment, via host filtration: can be concentrated by up to 10^4^ compared to surrounding water	Gut; unclear whether true gut microbionts, or transient occupants concentrated from food and water
Shellfish: abalone, *Haliotis*	Reviewed in Sawabe (2006)	*V. haliotis*	Culture	*In situ* enrichment: ~70% of culturable gut bacteria; reproducibly specific association	Gut; may contribute to host seaweed digestion via alginolytic activity
Squids: Sepiolid (*Euprymna scolopes*) and loligonoid	Reviewed in Ruby and Lee (1998); Stabb (2006)	*V. fischeri*	Culture, molecular	Exclusive light organ symbiotes	Bioluminescent symbiotes of nutrient-rich light organ. Colonize immature squid; in mature fish, are expelled and recolonize daily, outcompeting nonsymbiotes
*Vertebrates*					
Bluefish	Newman et al., 1972: New York, USA	*Vibrio* spp.	Culture	*In situ* enrichment: can dominate gut bacteria	
Coral reef fishes, incl. surgeonfish *Acanthurus nigricans*, parrotfish *C. sordidus*, snapper *Lutjanus bohar*	Sutton and Clements, 1988; Smriga et al., 2010: Palmyra Atoll, northern Pacific	Spp. including *V. agarivorans, coralliilyticus, fortis, furnissii, ponticus, qinhuangdaora, nigripulchritudo; Photobacterium* spp.	Culture, molecular	*In situ* enrichment: can dominate gut bacteria, according to both culturing and molecular methods. Molecular quantification: 10% of *A. nigricans* gut community, 71% of *C. sordidus*, 76% of *L. bohar*	Gut; unclear whether true gut microbionts, or transient occupants ingested from food (i.e., coral, for parrotfish) and water
Flashlight fishes (Anamalopidae) and anglerfishes (Ceratioidei)	Haygood and Distel, 1993	Novel *Vibrio* spp.	Molecular	Exclusive light organ symbiotes	Bioluminescent symbiotes of nutrient-rich light organ
Flatfishes incl. Rajidae skate, lemon sole *Microstomus kitt*, turbot *Scopthalmus maximus*	Liston, 1957: Scotland, UK; Xing et al., 2013: fish farm, China	Spp. incl. *V. cholerae, parahaemolyticus, cholerae; Photobacterium* spp.	Culture, molecular	*In situ* enrichment: Can dominate gut bacteria, according to both culturing (35–74%, *M. kitt*) and molecular (~80%, *S. maximus*) methods	Gut; unclear whether true gut microbionts, or transient occupants ingested from food and water
Jackmackerel *Trachurus japonicus*	Aiso et al., 1968: Japan	*Vibrio* spp.	Culture	*In situ* enrichment: 27% of stomach culturable bacteria, 100% of intestine	Gut; unclear whether true gut microbionts, or transient occupants ingested from food and water
Salmonidae, incl. pink salmon *Onchorhynchus gorbuscha*, chum salmon *O. keta*, sockeye salmon *O. nerka*, Chinook salmon *O. tshawytscha*	Yoshimizu and Kimura, 1976: Japanese coast, East Bering Sea	*Vibrio* spp.	Culture	*In situ* enrichment: dominate gut bacteria of saltwater-dwelling (but not freshwater) salmonids; on average represent 69% of saltwater gut community	Gut; unclear whether true gut microbionts, or transient occupants ingested from food and water
Sea bream *Pagrus major*, *Acanthopagrus schlegeli*	Muroga et al., 1987: Japan	*Vibrio* spp.	Culture	*In situ* enrichment: ~45% of culturable gut bacteria	Gut; unclear whether true gut microbionts, or transient occupants ingested from food and water

### Associations with plants

Vibrio survival is enhanced in association with certain freshwater and estuarine plants (Table 1). Plant hosts can provide nutrition (Andrews and Harris, 2000) and the opportunity to form predation-resistant biofilms (Matz et al., 2005), and have been postulated to modulate unfavorably cold temperatures as well (Criminger et al., 2007). Two freshwater aquatic plants have been observed to support both *in situ* enrichment (in freshwater bodies of Bangladesh) and *in vitro* survival advantage for *V. cholerae*: duckweed, *Lemna minor* (Islam et al., 1990b), and water hyacinth, *Eichhornia crassipes* (Spira et al., 1981), with preference for roots of the latter. Concentration on *E. crassipes* roots may indicate that root exudate is a particularly rich nutritional source, but may also be an artifact of the fact that the roots represent the greatest area exposed to water, and hence to inoculation by planktonic *Vibrio*. By contrast, duckweed's minimal structure, lacking stem or developed leaves, means that almost the entire plant is in contact with the water and thus available for inoculation.

Among estuarine plants, nitrogen-fixing representatives of several *Vibrio* taxa—including *V. diazotrophicus*, *V. natriegens*, *V. cininnatiensis* (Urdaci et al., 1988), and *V. parahaemolyticus* (Criminger et al., 2007)—appear to be noteworthy members of the rhizosphere, given that they represent more than half of the culturable diazotrophs associated with the dominant marsh grasses *Spartina* sp. and *Juncus roemerianus* (Bagwell et al., 1998; Larocque et al., 2004), and the herb *Salicornia virginica* (Bergholz et al., 2001; Criminger et al., 2007). While this numerical dominance may reflect culturing bias, later molecular studies of the *S. alterniflora* rhizosphere confirmed that vibrios (not taxonomically resolved below the level of the family) are stable constituents of the community (Lovell et al., 2008), with little seasonal fluctuation (Gamble et al., 2010). Nitrogen fixation thus appears to be an effective strategy supporting *Vibrio* survival in the anaerobic rhizosphere, demonstrating the ecological breadth granted by vibrios' facultatively anaerobic metabolism.

### Associations with microalgae and filamentous cyanobacteria

While early culture-based studies have demonstrated numerical dominance of vibrios on phytoplankton surfaces compared to surrounding water, e.g., Simidu et al. (1971), little is known about direct, physical associations with specific phytoplankton. Algal cells represent a nutritional opportunity in that they often excrete a high proportion of their photosynthetically fixed carbon, thereby creating a diffusive sphere (the phycosphere) around them, with elevated organic carbon concentration compared to the bulk (Paerl and Pinckney, 1996). However, *in vitro* survival advantage and persistence have been thus far been demonstrated only for *V. cholerae* in physical association with two microalgae: with the filamentous freshwater green alga *Rhizoclonium fontanum* (Islam et al., 1989), and inside the mucilaginous sheath of *Anabaena* sp. cyanobacteria under both freshwater (Islam et al., 1990a, 1999) and saline conditions (Ferdous, 2009) (Table 1).

Recent work has illuminated mechanistic details of the *V. cholerae* association with *Anabaena*, which may follow the canonical model of symbioses between heterotrophic bacteria and nitrogen-fixing freshwater cyanobacteria. In such associations, heterotrophs locate their hosts via chemotaxis and benefit from rich cyanobacterial exudate (Paerl and Gallucci, 1985). In return, their oxidative metabolism both relieves oxygen inhibition of nitrogen fixation (which would otherwise limit rapid algal growth), and generates carbon dioxide for photosynthetic assimilation (Paerl and Gallucci, 1985). For *V. cholerae*, chemotactic preference for components of the Anabaena mucilaginous sheath has been demonstrated (Mizanur et al., 2002). Furthermore, investigators have shown that both chemotaxis to and survival on *Anabaena* depend on *V. cholerae*'s expression of mucinase (Islam et al., 2002, 2006). The exact role of mucinase has yet to be defined, but activity of secreted mucinase might liberate from mucus the relevant chemotactic attractants, aid colonizing *Vibrio* in physical penetration of the mucilage, and/or convert mucilage to nutritive compounds supplementary to the cyanobacterial exudate.

### Associations with macroalgae

Numerous studies have shown that vibrios are one of the most abundant culturable constituents of macroalgal communities (Table 1): a recent meta-analysis of 161, predominantly culture-dependent macroalgal-bacterial studies determined that vibrios on average comprised 10% of these communities (Hollants et al., 2013), with 28, 28, and 44% of them found on brown, green, and red macroalgae, respectively. While no molecular studies have yet quantified *Vibrio* within macroalgal communities, numerical enrichment of culturable vibrios has been demonstrated for the brown algae *Ascophyllum nodosum* (Chan and McManus, 1969), and *Laminaria longicruris* (Laycock, 1974); the red algae *Hypnea* sp. (Lakshmanaperumalsamy and Purushothaman, 1982), *Polysiphonia lanosa* (Chan and McManus, 1969), and *Porphyra yezoensis* (Duan et al., 1995); and the green algae *Chaetomorpha* sp. (Lakshmanaperumalsamy and Purushothaman, 1982), *Enteromorpha* sp. (Lakshmanaperumalsamy and Purushothaman, 1982), and *Ulva pertusa* (Duan et al., 1995). For *V. cholerae*, *in vitro* survival advantage has been shown on the green algae *Ulva lactuca* and *Enteromorpha intestinalis* and the red alga *Polysiphonia lanosa* (Islam et al., 1988).

As mentioned above, vibrios can metabolize many algal polysaccharides; they have furthermore been implicated in several other biological activities facilitating symbiosis with macroalgal hosts. These include antagonism directed toward potential bacterial or algal competitors for host surface area (Dobretsov and Qian, 2002; Kanagasabhapathy et al., 2008), developmental morphogenic effects on *Ulva pertusa* (Nakanishi et al., 1996), and stimulation of spore germination for *Ulva* sp. (Patel et al., 2003; Tait et al., 2005). Hence multiple lines of evidence point to significant *Vibrio* association with *Ulva* sp. (enrichment, survival, morphogenesis and spore modulation) and *Polysiphonia* sp. (enrichment, survival) in particular.

### Associations with animals

*Vibrio* interactions with animals include both specific, stable symbioses, and less well-defined associations (Table 2). Stable symbioses have been described for luminescent *V. fischeri* (*Aliivibrio*) with sepiolid squids (*Euprymna scolopes*) and loligonoid squids (Ruby and Lee, 1998), and for various luminescent *Vibrio* with flashlight fishes (Anamalopidae) and anglerfishes (Ceratioidei) (Haygood and Distel, 1993). The dynamics of the *V. fischeri*-*Euprymna symbiosis* have been particularly well-explicated: *V. fischeri* from surrounding waters colonize the developing squid light organ, successfully outcompeting non-symbionts in this process, which triggers a developmental program in the host. Once established, the symbionts undergo daily cycles of expulsion and regrowth (Ruby and Lee, 1998; Stabb, 2006). Thus the symbiosis regularly seeds the water column, such that luminous *V. fischeri* are enriched in the water surrounding *E. scolopes* (Ruby and Lee, 1998). This expedites continual recolonization of immature squid, which is likely further facilitated by *V. fischeri* chemotaxis toward squid mucus (DeLoney-Marino et al., 2003).

Some *Vibrio* have also been deemed facultative intracellular symbionts of *Acanthamoeba* protozoa: *Vibrio cholerae* O1 and O139, and *Vibrio mimicus* (Abd et al., 2005, 2007, 2010; Sandström et al., 2010). These vibrios can replicate intracellularly for at least 14 days without affecting host health, at least in nutrient-replete artificial medium, and have been observed in both cytoplasm and cysts of the protozoa. Like several other microbial taxa, then, most famously the pathogen *Legionella* (Rowbotham, 1980), vibrios appear capable of evading *Acanthamoeba* endocytosis to shelter intracellularly. Thus they gain protection from antibiotics (Abd et al., 2005, 2007, 2010), predation, and perhaps other adverse conditions, e.g., cold temperatures. Still to be investigated are the questions of why some *Acanthamoeba* cells encyst their *Vibrio* inhabitants while others do not; why the *Vibrio* do not appear to be detrimental to host survival; and how often *Vibrio* might be released following host lysis, or even actively ejected, thus returning to the water column. Moreover, all studies of the *Vibrio*-*Acanthamoeba* relationship have been experimental: *in situ* surveys are necessary to establish the environmental relevance of this potential symbiosis, and assess any effects on *Vibrio* population dynamics.

Vibrios may be neutral or benign inhabitants of coral hosts: they have been shown to comprise a significant portion of the mucus-dwelling bacterial community of healthy corals (e.g., Koren and Rosenberg, 2006; Kvennefors et al., 2010), being able to subsist on coral mucus as their sole carbon and nitrogen source (Sharon and Rosenberg, 2008). *V. splendidus*, for example, constituted 50–68% of clone libraries derived from *Oculina patagonica* coral mucus, but was scarce in the coral tissue itself (Koren and Rosenberg, 2006). Moreover, nitrogen-fixing *Vibrio* representatives, primarily *V. harveyi* and *V. alginolyticus*, have been found to dominate the culturable diazotrophs of the coral *Mussimilia hispida* (Chimetto et al., 2008), and likely share fixed nitrogen with either or both coral and zooxanthellae. Evidence also suggests immune interaction between *Vibrio* and coral hosts: adaptation of *Vibrio* commensals to coral antimicrobials has been suggested by significant antibiotic-resistance gene cassette content of their integrons (Koenig et al., 2011), while one *V. harveyi* coral isolate has been found to help defend its host by inhibiting colonization by a pathogen (Krediet et al., 2013).

In freshwater habitats, *V. cholerae* have been found to proliferate on egg masses of the abundant, widely distributed chironomid midges (Broza and Halpern, 2001; Halpern et al., 2008). These egg masses are embedded in thick, gelatinous material, which *V. cholerae* can use as a sole carbon source (Broza and Halpern, 2001); their degradation of the gelatinous matrix via secreted hemagglutinin/protease appears to be the primary cause of egg mass disintegration (Halpern et al., 2003). Accordingly, Halpern et al. (2006) were able to show correlations of chironomid egg mass with the abundance of attached *V. cholerae*, although they have not yet investigated any correlation of *V. cholerae* dynamics in the surrounding aquatic environment.

Zooplankton, primarily estuarine copepods such as *Acartia* and *Eurytemora*, have been investigated as a major reservoir of *V. cholerae* in particular, but while attachment has been demonstrated, it remains unclear whether the association is specific, and whether attached vibrios are consistently enriched compared to surrounding waters. Individual copepods have been shown to be able to host up to 10^5^ *V. cholerae* cells (Colwell, 1996; Mueller et al., 2007), with preference often shown for attachment to the oral region and egg sac (next to the anal pore)—that is, regions offering close access to host exudates (Huq et al., 1983, 1984). Culture-based studies have detected enriched *Vibrio* occurrence on copepods compared to the surrounding water column (e.g., Simidu et al., 1971; Sochard et al., 1979), and one culture-based study showed *Vibrio* dominance of wild copepods' surface- and gut-attached bacterial communities (Sochard et al., 1979). However, other studies, both *in vitro* and *in situ*, have observed *V. cholerae* remaining predominantly free-living in the presence of copepods (Worden et al., 2006; Neogi et al., 2012) or attaching with greater preference to phytoplankton (Tamplin et al., 1990). Additionally, one culture-independent environmental study detected greater concentrations of Vibrio, including *V. cholerae*, in water compared to zooplankton (Heidelberg et al., 2002a,b). Perhaps such variability of association with copepods helps explain the difficulty in detecting correlated *Vibrio*-copepod dynamics, as mentioned above in the section Environmental Correlates of *Vibrio* Presence and Abundance.

Other uncertainties regarding *Vibrio* association with copepods exist. There is a lack of quantitative evidence demonstrating long-term proliferation of copepod-attached *Vibrio*: existing studies assessing survival advantage of *Vibrio* cultured with copepods have only demonstrated increased abundance of *Vibrio* in surrounding water, without monitoring attached abundance (Huq et al., 1983, 1984). Finally, it is not clear whether vibrios prefer colonizing live or dead copepods. While several *in vitro* studies have noted *V. cholerae* attachment preference for dead or detrital copepods (Huq et al., 1990; Tamplin et al., 1990; Mueller et al., 2007), one study instead observed survival advantage only upon association with live copepods, and found little attachment to dead copepods (Huq et al., 1983). Perhaps this question could be resolved by investigating from which part(s) exactly of the copepod vibrios derive nutrition: from oral/anal exudates or gut contents of actively feeding copepods, from degradation of the chitinaceous exoskeleton which for live copepods is protected by a waxy epicuticle that resists attachment (Tarsi and Pruzzo, 1999), or from degradation of other copepod detritus. In addition, variable host traits such as immune defenses, age, and time since molting or death (which likely affect epicuticle condition) should be taken into account. As of yet, evidence of association with live copepods as an ecological specialization has been demonstrated for only one *Vibrio* sp. nov. (F10) (Preheim et al., 2011a).

In addition, zooplankton other than copepods may represent potential *Vibrio* hosts as well. Kirschner et al. (2011) found cladoceran *Diaphanosoma mongolianum* to enhance growth more than the copepod *Arctodiaptomus spinosus* in microcosm experiments; when cladocerans were added, they enhanced the growth of *V. cholerae* strains in the surrounding medium relative to controls where cladocerans were excluded, while copepods did not. In addition, the number of cells attached to cladocerans per individual was on average 100 times higher than on copepods. When a back-of-the-envelope calculation is done to consider whether *V. cholerae* is enriched on zooplankton, however, we find that they are not, even on cladocerans; from six microcosms, 10^5^–10^7^ cells were estimated attached and 10^6^–10^7^ cells not attached, a result suggesting that cladocerans might enhance overall growth with frequent dispersal, rather than supporting exclusively attached growth.

For other animals in which *Vibrio* have been found to be abundant—fish, and shellfish—it has not yet been determined whether vibrios form specific, lasting associations as gut microbiota, or are merely transient occupants, temporarily proliferating on favorable nutrients until excreted or otherwise detached. In marine fish, numerous studies, both culture-dependent and -independent, have demonstrated that *Vibrio* are major gut inhabitants, often dominating the community, and hence are substantially enriched compared to surrounding seawater. Surveyed fish include flatfish (Liston, 1957; Xing et al., 2013), jackmackerel (Aiso et al., 1968), bluefish (Newman et al., 1972), salmonids (Yoshimizu and Kimura, 1976), sea bream (Muroga et al., 1987), and various coral reef fishes (Sutton and Clements, 1988; Smriga et al., 2010). Notably, *Vibrio* abundances often appear comparable between culture-based and -independent studies: e.g., 35–74 and 83.4%, respectively, of flatfish inhabitants (Liston, 1957; Xing et al., 2013). The ability of *Vibrio* representatives to resist low pH and bile supports their survival within the fish gut (Yoshimizu and Kimura, 1976). Whether food or water intake is the greater source of inoculation is an open question: some studies have found a strong effect of food source on gut *Vibrio* composition (e.g., Grisez et al., 1997), whereas others found a stronger influence of *Vibrio* representation in the water column (e.g., Blanch et al., 2009). Conversely, *Vibrio* content of the fish gut has also been shown to be responsible for increasing *Vibrio* abundance in surrounding water when fish were introduced into a tank that did not otherwise support *Vibrio* growth, demonstrating significant excretion of viable cells from the fish gut (Sugita et al., 1985). Hence, regardless of length of association, the fish gut appears to represent a favorable refuge where *Vibrio* can rapidly proliferate, prior to being released again to the water column. Indeed, the bioluminescence of marine microbes, including many vibrios, has been suggested to be an adaptation encouraging fish ingestion: fish preferentially predate zooplankton that are glowing after having grazed bioluminescent *Photobacterium* (Zarubin et al., 2012).

Among shellfish, high *Vibrio* abundance has been reported on surfaces and in tissues of hosts including oysters (e.g., Murphree and Tamplin, 1995; Froelich and Oliver, 2013), abalone (Sawabe, 2006), and blue crabs (Davis and Sizemore, 1982), with uptake and population dynamics particularly well-documented for *V. vulnificus* in association with oysters (Froelich and Oliver, 2013). *V. haliotis* has been suggested to stably associate with gut of the herbivorous *Haliotis* abalone on the basis of reproducibly specific occurrence: it has never been isolated from other seaweed-consuming invertebrates (reviewed in Sawabe, 2006). Being alginolytic, *V. haliotis* has also been suggested to aid its host's digestion of algal polysaccharides (Sawabe, 2006). Otherwise, it is not clear whether copious *Vibrio* representation might solely be the result of non-specific uptake from food or water, particularly for filter-feeding shellfish, whose highly efficient filtration has been reported to increase *Vibrio* concentrations by up to 4 orders of magnitude in oysters compared to surrounding waters (Froelich and Oliver, 2013). Furthermore, filter feeders can produce copious amounts of mucus, which rapidly and efficiently removes associated microbes, so that their turnover may be high. Consequently, it is challenging to prove specific association on the basis of abundance. In the next section, we will review a metapopulation study that more explicitly addresses the problem of assessing *Vibrio* host specificity by analyzing population structure across and within macroinvertebrate hosts. Future application of the approach described could help to resolve the question of whether *Vibrio* colonization of animal hosts like fish and crabs is specific, or driven more by indiscriminate uptake from the water column.

### Population dynamics associated with macroinvertebrate hosts

In a metapopulation study by Preheim et al. (2011a), relative abundances of *Vibrio* groups were compared across different shellfish and parts of shellfish. The study found that macroinvertebrates do not appear to be a strongly selective habitat for vibrios, when contrasted to preceding metapopulation studies of the water column, where differential associations of genotype clusters revealed ecologically distinct populations (described in detail in the section Using Ecology to Define Cohesive Populations). When different body parts of mussels and crabs were sampled by Preheim et al. (2011a), little host preference was evident, and the diversity and frequency of populations (identified by multi-locus sequence analysis) resembled that in water samples. For example, *V. splendidus* represented the dominant population in the water and on both animals. For mussels, which can retain particles when filter feeding (Vahl, 1972), the similarity between water column and animal-associated populations was particularly high, and there appeared to be relatively little difference when gills, stomach and gut walls and contents were compared. This was interpreted as population assembly being largely driven by filter-feeding activity, as was posited in the section above. In contrast to mussels' highly uniform population structure across individual hosts, crabs showed high variance in associated *Vibrio* populations, although composition across individuals' body parts was still similar to that in the water column. What causes the high variance among individual crabs is not known, although there was some evidence suggesting that they may be inoculated by food items, which could be of variable composition given their scavenging lifestyle.

The apparent lack of specificity for the animals was surprising considering that ecological theory predicts that habitats that are long-lived and stable compared to the colonizing species should be dominated by specialists (Kassen, 2002). Yet with regard to mussels and crabs as habitats, vibrios appear to be generalists whose population dynamics may be determined by direct inoculation from the water or via food items (Preheim et al., 2011a). A similar dynamic has recently been suggested to drive *V. vulnificus* accumulation in oysters (Froelich et al., 2010). These can only retain larger particles when filter feeding, and hence enrich pathogenic ecotypes of *V. vulnificus* that are particle-associated as compared to ecotypes that are predominantly free-living.

Overall, these studies demonstrate that colonization may be a complex process strongly influenced by dispersal. In contrast to water column populations, which showed varying degrees of specificity toward microhabitats (e.g., organic particles, zooplankton), *Vibrio* populations on larger invertebrates (mussels and crabs) showed little specificity either for host or host body parts. Whether similar patterns exist for other animals remains unknown; it will be valuable to test fish to determine whether their *Vibrio* inhabitants are true gut microflora. The above studies stress the importance of taking into account potential *Vibrio* sources, i.e., water and food, when assessing host association. For example, *V. splendidus* was the dominant population on both crabs and mussels, and on particles in the water column; had only mussels been sampled, *V. splendidus* may have appeared to have been a mussel specialist. Such erroneous conclusions can be avoided by “mass balancing” populations in a particular location by determining their frequency across different microhabitats or patches that are potentially connected by migration.

## *Vibrio* proliferation in the water column

Ocean water is a heterogeneous landscape of varying ecological opportunities on small scales, with a highly patchy distribution of resources that may represent microhabitats for vibrios. Some of these are hotspots of soluble organic material, which originates from exudates or excretions of larger organisms, while others are particulates of various origins. For example, as mentioned above, algal cells exude a zone of enriched organic material (Bell and Mitchell, 1972; Paerl and Pinckney, 1996). Several other processes can also generate ephemeral patches of dissolved nutrients, and it is likely that many bacteria, including vibrios, can chemotax toward these and take advantage of the elevated nutrient concentrations (e.g., for vibrios, Sjoblad and Mitchell, 1979; Mizanur et al., 2001, 2002). In addition, diverse processes are responsible for the formation of suspended particulate organic matter that can be colonized and degraded by bacteria. This includes dead biomass of small planktonic organisms, fecal pellets, and aggregates (marine snow) formed from polymers and other, smaller particles.

This section will address two main subjects, both seeking to situate *Vibrio* within the marine water column. Here, we will first review both experimental and environmental evidence that blooms of *Vibrio* can and do occur, despite their typically low representation in marine assemblages. Second, we will review the evidence for proliferation of *Vibrio* in the planktonic, free-living phase, expanding the view of their niche range beyond the longstanding proposition that their lifestyle is predominantly attached.

### *Vibrio* blooms

Thompson and Polz (2006) summed up three key *Vibrio* traits supporting the ability to bloom on sporadic nutrient pulses: *Vibrio* can (1) survive long-term under resource-limited conditions, as indicated by continued respiratory activity in mesocosms (Ramaiah et al., 2002; Armada et al., 2003); (2) recover from starvation and grow rapidly in response to substrate pulses, enabled by maintenance of high ribosome content (Hood et al., 1986; Flärdh et al., 1992; Kramer and Singleton, 1992; Eilers et al., 2000; Pernthaler et al., 2001); and (3) actively seek out nutrient patches via chemotaxis (Bassler et al., 1991; Yu et al., 1993), including under starvation conditions (Gosink et al., 2002; Larsen et al., 2004).

*Vibrio* proliferation on natural dissolved resources alone has been experimentally demonstrated by rapid growth of inocula in mesocosms or microcosms of filtered water from algal blooms. *V. cholerae* strain N19691 grew at a rate of up to 2.6 d-1 in dinoflagellate (*Lingulodinium polyedrum*) bloom water (Mouriño-Pérez et al., 2003), and up to 1.73 d-1 in water from a dense picophytoeukaryote and dinoflagellate bloom, surpassing the 0.76 d-1 average growth rate of the separately incubated native bacterial assemblage (Worden et al., 2006).

Experiments have furthermore demonstrated conditions where algal resources were sufficient for *Vibrio* to overcome competition and/or grazing pressure. Taking competition into account, but in the absence of predation, strains of both *V. cholerae* and *V. vulnificus* have been shown capable of increasing in relative abundance when in direct competition with the total bacterial community for filtered homogenate of a cyanobacteria bloom (dominated by *Nodularia spumigena*) (Eiler et al., 2007). Meanwhile, *V. cholerae* N19691 has been shown to overcome substantial protozoan grazing when proliferating on filtrate of a particularly dense algal bloom (Worden et al., 2006). Ample algal dissolved organic material may have permitted this *V. cholerae* growth by relieving resource competition, as the *V. cholerae* inocula grew at the same rate with or without the whole bacterial community filtered out from their bloom-water amendments. Similarly, an analysis of *Vibrio* dynamics sampled from the Arabian Sea suggested that algal resource supply can be a more significant control on *Vibrio* abundance than predation, enabling rapid turnover (Asplund et al., 2011).

Reinforcing these experimental findings, (Gilbert et al., 2012) observed an explosive *Vibrio* bloom in the environment, demonstrating that their potential for rapid growth is indeed relevant in the context of a full marine community. In 1 month, a single *Vibrio* sp., otherwise comprising only 0–2% of total rRNA genes, grew to constitute 54% of the community—the largest bloom of any bacterial group observed over the course of a 6-year time series. Furthermore, there was a correlated bloom of the diatom *Chaetoceros compressus*, itself typically rare within the phytoplankton community. Hence, nutrients exuded by the unusually proliferating diatom taxon may have sparked the *Vibrio* bloom, whether by specifically appealing to the species' metabolic palate, relieving resource competition, diluting protozoan grazing pressure by stimulating rapid growth of the surrounding bacterial community, or some combination of the three. Luminescent *Vibrio* blooming in association with algae have even been suggested to be responsible for the phenomenon dubbed “milky seas,” where significant stretches of surface water are rendered white with bioluminescence (Lapota et al., 1988; Nealson and Hastings, 2006); one recent case was expansive enough (>17,700 km^2^) to be detectable by satellite. Whether such bloom events are rare remains unknown due to currently infrequent sampling and lack of time series; however, the observations cited above provide evidence that *Vibrio* are capable of rapid growth in the environment.

### The evidence for a planktonic, free-living lifestyle

The two mesocosm/microcosm studies discussed above (Mouriño-Pérez et al., 2003; Worden et al., 2006), both furnish evidence that vibrios can thrive while free-living. Mouriño-Pérez et al. (2003) demonstrate the ability of a *V. cholerae* strain to flourish purely on dissolved compounds derived from an algal bloom. Even more strikingly, Worden et al. (2006) observed *V. cholerae* N19691 remaining free-living in four out of their five seawater mesocosm experiments: one initiated from non-bloom seawater, and the other three initiated from seawater collected during distinctly different phytoplankton blooms. Notably, in two of these four experiments, *V. cholerae* attachment to cohabiting copepods was assessed and found to be insignificant (e.g., <1 *V. cholerae* cell found per copepod, averaged over a sampling of 10 copepods, in one of the experiments). This stands in contrast to the theory that *V. cholerae* preferentially attach to copepods, as discussed above in the section on animal associations. In the remaining experiment of (Worden et al., 2006), by contrast to the mesocosms in which *Vibrio* remained free-living, the *V. cholerae* inoculum was initially almost entirely free-living, but, as bloom decay progressed and algal detrital particles increased in size, the population became almost entirely particle-attached, presumably in response to nutrient limitation.

The factors determining whether *Vibrio* remain free-living versus particle-attached are still unknown, but both environmental and genetic determinants could come into play. Past studies have demonstrated effects of temperature, pH, ion concentration, and starvation state (Hood and Winter, 1997); salinity (Kumazawa et al., 1991; Hsieh et al., 2007); and growth-stage-dependent chitin content of diatom cell walls (Frischkorn et al., 2013) on *Vibrio* attachment. Perhaps encounters with relevant biological compounds, e.g., a specific algal cell wall component or polysaccharide, might also trigger lifestyle changes. Even less is known about the genetic mechanisms, diversity, and dynamics underlying vibrio lifestyle association; this remains a rich field of inquiry. For example, Shapiro et al. (2012) recently discovered genomic patterns underlying the ongoing ecological differentiation of two *V. cyclitrophicus* populations: the population with preference for association with larger particles possessed genes for attachment and biofilm formation that were absent from the preferentially free-living population. Such evidence of genetic bases for habitat specificity will provide invaluable insights into selective pressures exerted by different marine microhabitats.

The findings described above suggest great flexibility in *Vibrio* lifestyle, permitting many lines of attack on marine substrates, with different ecological implications for vibrios' dynamics in the water column. For example, biofilm attachment on particulate resources can decrease susceptibility to protozoan predation (Matz et al., 2005), while association with larger particles might increase probability of ingestion by macrofaunal predators, which could in turn facilitate rapid proliferation and dispersal, as discussed above in the section on fish associations. Given vibrios' possibilities for rapid growth and association with diverse marine niches and resources, their impacts on marine nutrient cycling and trophic structure might be much greater than previously believed. Understanding their dynamics will help to elucidate these fundamental marine processes, as well as *Vibrio*-specific models of pathogen persistence and transmission.

## Using ecology to define cohesive populations

The studies summarized above suggest potential for association of vibrios with plants, algae, and animals as well as growth response to specific classes of particulate and dissolved organic matter; however, they have targeted primarily a single, taxonomically defined species, leaving several important questions unanswered. First, do such taxonomic species correspond to ecologically cohesive units, i.e., do they comprise several ecologically distinct populations or should they be merged with others to form one ecologically cohesive population? Second, if we can define such populations, do these partition resources or compete with each other? Finally, are vibrios primarily ecological generalists or specialists?

A series of studies explored to what extent ecologically coherent groups of vibrios could be distinguished by determining the distribution patterns of genotypes among different potential microhabitats in the coastal ocean (Hunt et al., 2008a; Preheim et al., 2011a,b; Szabo et al., 2013). Initially, this was done by isolation of vibrios from four consecutive size fractionations of ocean water, collected in the spring and fall, to distinguish free-living and attached genotypes (Hunt et al., 2008a). The rationale of this sampling scheme was that different types of microhabitats (e.g., organic particles of various origin, zoo- and phytoplankton) have characteristic size spectra and hence will be enriched in a specific size fraction. Consequently, bacteria specifically associated with a microhabitat should be enriched in the same specific size fraction. Further, because ecological associations may evolve on faster time scales than rRNA genes, isolates were also characterized at higher genotypic resolution using several protein coding genes in a multilocus sequence analysis (MLSA) scheme, to better capture the eco-evolutionary dynamics of environmental populations. Because of the complexity of the data, a statistical clustering algorithm (AdaptML) was developed that allows identification of groups of related genotypes with distinct and characteristic distributions among the sampled parameters (size fractions and seasons) (Hunt et al., 2008a).

The analysis of >1000 isolates identified a large number of genotypic clusters with clear microenvironmental preferences, consistent with the notion of an ecological population (Hunt et al., 2008a). Seasonal differentiation was particularly strong, with little overlap between spring and fall samples, supporting the observed significant correlation of some species to temperature discussed in above sections. The study also revealed that several populations appear free-living or predominantly free-living, again supporting the notion that vibrios can pursue, at least temporarily (e.g., during a bloom), unattached lifestyles. Most populations, however, displayed various preferences for size fractions enriched in different types of organic particles or zoo- and phytoplankton. For example, *V. calviensis* appeared almost entirely free-living, while *V. alginolyticus* had significant representation in both the free-living and large-particle fractions, and *V. fischeri* occurred on small and large particle size fractions. Most strikingly, *V. splendidus* was broken up into several, very closely related populations with distinct distributions. Overall, 25 distinct populations could be identified in the two seasonal samplings. (Hunt et al., 2008a), demonstrating the fine-scale resource partitioning co-existing vibrios are engaged in.

To what extent does the commonly used rRNA marker gene resolve these populations? The *V. splendidus* example and several others demonstrate that at least some ecologically distinct genotypic clusters may not be resolved by rRNA analysis and do require high resolution protein-coding genes to identify genotypic clusters whose environmental distributions can be assessed (Preheim et al., 2011b; Shapiro et al., 2012). Most populations, however, were manifest as deeply divergent protein-coding gene clusters (Hunt et al., 2008a) that correspond to microdiverse rRNA gene clusters previously postulated to represent ecological populations (Acinas et al., 2004). Although overall reassuring for rRNA gene-based environmental surveys, variable performance of marker genes is expected since they are slowly evolving and may not capture populations at early stages of divergence (Shapiro et al., 2012).

Additional studies carried out at the same coastal site refined the habitat resolution for several populations, allowed identification of ecological generalists and specialists, and also demonstrated reproducible associations (Preheim et al., 2011a,b; Szabo et al., 2013). The actual microhabitat of several attached populations was identified by hand-picking under the microscope visually identifiable types of particles and zooplankton (Preheim et al., 2011a,b). This revealed high habitat specificity for several populations while others occurred more broadly, indicating different levels of ecological specialization. For example, *V. breoganii* occurred on algal derived detritus while a not yet formally described species (*Vibrio* F10) was highly specific for living zooplankton. On the other hand, *V. crassostreae* was associated with both zooplankton and algal detritus. Metabolic potential in these species, measured by growth assays and comparative genomics, reflects these associations. Both *V. breoganii* and *V. crassostreae* are able to exploit alginate, a brown algal cell wall component, as the sole carbon source, yet the algae-associated *V. breoganii* has acquired the ability to grow on the algal storage polysaccharide laminarin but has lost the ability to grow on chitin, a trait ancestral to vibrios (Hunt et al., 2008b). Moreover, such high specificity for algal derived material was unexpected for vibrios, which are reputed to be animal associated, and supports the evidence provided above that vibrios encompass algal specialists.

A recent study that attempted to reproduce the original size fractionation of ocean water collected at a similar time point, but 3 years after the initial sampling, showed that population structure was preserved for many of the originally detected populations, but also revealed populations as dynamic and environmentally responsive entities (Szabo et al., 2013). For example, *V. breoganii*, *V. crassostreae*, and *V. splendidus*, which range in ecological specialization from specialist to generalist, had highly reproducible distributions indicative of similar habitat associations. The study, however, also showed that several populations were nearly absent in either of the samplings, possibly due to the lower frequency of their habitat in the water samples. Moreover, some populations had shifted distributions among the size fractions. This was the case for a recently diverged population of *V. cyclitrophicus* that was associated with larger particles or organisms in the first study, but was highly represented in the free-living fraction in the second sampling. It was hypothesized that this shift represented a population expansion following a diatom bloom because the relative frequency of *V. cyclitrophicus* increased coincident with a shift from a copepod- to a diatom-dominated eukaryotic plankton community. Similarly, bloom dynamics, as have previously been observed for total vibrios in the water column, may cause the variable representation of several additional populations. Overall, the comparison of the two studies supports highly predictable population-habitat linkage but also provides additional support for the notion that vibrios may be subject to rapid population expansions or blooms in response to often overlooked or unknown environmental factors.

## Populations as ecological, genetic, and social units

Populations as defined here are genotypic clusters (evident by MLSA) that act as ecologically cohesive units, i.e., their ecology is more similar within the cluster than between. Defining populations in this way has afforded the opportunity to test the hypothesis that, akin to sexual eukaryotes, gene flow boundaries across such clusters are strong enough for adaptive genes or alleles to spread in a population-specific manner. A population genomic analysis of two very recently diverged populations of *V. cyclitrophicus*, which are ecologically distinct but remain >99% similar in average nucleotide composition across their genomes, showed that specific genome regions have swept each of the two populations (recently reviewed by Polz et al., 2013). Moreover, annotation of these genome regions as well as behavioral and growth analysis suggest that these genome regions are adaptive for differential lifestyles (Shapiro et al., 2012; Yawata et al., 2014).

A second study showed that ecologically defined populations may also act as social units. This was evident in a test for potential of antagonistic interactions mediated by antibiotics between individuals from different ecological populations of vibrios (Cordero et al., 2012). Because of higher niche overlap among close relatives, it was expected that antagonism be more advantageous if directed against members of the same population. In stark contrast, however, antagonism was primarily directed against members of other populations while members of the same population were resistant to antibiotics produced within their own populations. This suggests synergism on the population level, especially since multiple antibiotics were produced within each population but each only by relatively few members.

Overall, this research shows agreement between ecological, genetic, and social population structure and suggests that, in many ways, populations can be regarded as species-like units in the wild. Importantly, these units are non-clonal, and their genetic exchange and social structure suggest that populations frequently coexist and re-assemble on small-scale habitats.

## Conclusion

In this review, we examine what is known about *Vibrio* ecology at increasingly fine environmental and taxonomic scales, to reveal factors with potential for greater predictive and explanatory power for *Vibrio* dynamics.

We find that while bulk environmental variables are often inconsistent in their ability to explain variance in *Vibrio* abundances, at both the genus and species levels, temperature and salinity are usually the strongest abiotic correlates. Yet total *Vibrio* trends do not necessarily capture species-level trends, and thus it is necessary to monitor populations of interest directly to capture their dynamics. Correlations of species to specific plankton—like those of *V. cholerae* to dinoflagellate (Eiler et al., 2006), cladoceran (Kirschner et al., 2011), and rotifer (de Magny et al., 2011) taxa—can provide the bases for hypotheses of biological associations—as was demonstrated by Kirschner et al. (2011) for cladoceran the *D. mongolianum*. Further investigation is necessary to confirm reproducibility and biological significance of such correlations.

Indeed, the breadth of vibrios' metabolic and attachment abilities mean that they can appear quite generalist in their ecological associations, making it difficult to discern which relationships with other organisms are specific and stable, rather than simply the product of promiscuous attachment followed by proliferation. Among the diverse biological associations that we review, some may be true mutualisms, on the basis of vibrios exchanging benefits with their hosts. The symbioses of luminescent vibrios with certain squid and fish are well-attested, while possible symbioses with other organisms are suggested by potentially mutual metabolic exchange (salt marsh plants, cyanobacteria, corals), or *Vibrio* modulation of host processes like development and reproduction (macroalgae), and response to infection (corals). Notably, diazotrophy may facilitate relationships with both marsh plants and corals. In numerous other cases, vibrios may simply be taking advantage of hosts as nutrient sources, and perhaps only temporarily and opportunistically be associated with microalgae, zooplankton, fish, shellfish, and chironomid egg masses, or as intracellular occupants of protozoa. Of these, we argue that evidence points toward a particularly significant ecological impact of *Vibrio* interactions with algae, given the abundant laboratory and environmental observations of vibrios' ability to live on algal exudates - including blooms as free-living cells, a historically underappreciated *Vibrio* lifestyle. Nonetheless, much work remains to be done in resolving more specific *Vibrio*-algae associations.

In light of these studies, we have several recommendations. Previous surveys of *Vibrio* abundance are predominantly culture-dependent; going forward, molecular methods, such as fluorescent *in-situ* hybridization or quantitative PCR, can be used to gain less biased quantitative data. Such techniques also enable targeting of specific genotypic groupings, allowing better discrimination of pathogenic variants or ecologically meaningful populations than traditional taxonomic assays of species identity. Furthermore, to distinguish specialized association from incidental attachment, a “mass-balanced” approach is necessary: are vibrio enriched on a given microhabitat (e.g., a specific organic particle type or zooplankton) compared to the surrounding water? Or, is the habitat enriched in *Vibrio* compared to other habitats? This approach has provided support for many of the potential symbioses noted above, and enabled identification of specialist *Vibrio* populations, e.g., *V. breoganii* for macroalgae-derived material and *V*. F10 for zooplankton (Hunt et al., 2008a; Preheim et al., 2011a,b; Szabo et al., 2013). It provides a strong basis from which to proceed to more detailed and, ideally, mechanistic elucidation of *Vibrio* associations: for example, identifying chemotactic preferences for or proliferation on host or host exudates, or taking advantage of vibrios' genetic tractability to demonstrate dependence of an association on particular metabolic pathways.

When considering the question of to what extent environmental affiliations may be shared among or within *Vibrio* taxa, we also explore the shifting perspective on the nature of microbial groupings: recent work has moved toward discerning ecologically cohesive *Vibrio* populations, rather than relying on named species as the unit of inquiry. Pursuing this approach, whereby habitat associations are mapped onto genotypic clusters, has been successful in identifying ecological, genetic and social units among vibrios in the wild. We stress, however, that the initial identification of environment-genotypic cluster associations by the “mass-balanced” approach outlined above must be treated as a hypothesis of population structure to be further explored by more mechanistic investigation of, for example, dynamic habitat associations, biological interactions and gene flow boundaries. As demonstrated above, this approach has already helped to resolve apparently generalist *Vibrio* taxa into specialized populations and to identify mechanisms of how adaptive genes spread amongst nascent, ecologically differentiated populations. By sampling the environment at fine scales and molecularly characterizing associated *Vibrio*, we will gain a deeper understanding of the ways in which vibrios live in the environment. Such a population-based framework serves as a means of understanding the ecology of microorganisms in general.

## Author contributions

All authors conceived the structure and content, wrote the text as well as edited the manuscript in its entirety.

### Conflict of interest statement

The authors declare that the research was conducted in the absence of any commercial or financial relationships that could be construed as a potential conflict of interest.
